# Lattice‐Distortion‐Driven Reduced Lattice Thermal Conductivity in High‐Entropy Ceramics

**DOI:** 10.1002/advs.202501157

**Published:** 2025-03-20

**Authors:** Yiwen Liu, Yaming Fu, Fangchao Gu, Hulei Yu, Lei Zhuang, Yanhui Chu

**Affiliations:** ^1^ School of Materials Science and Engineering South China University of Technology Guangzhou 510641 China

**Keywords:** high‐entropy ceramics, lattice distortion, molecular dynamics, thermal conductivity

## Abstract

Lattice distortion and mass fluctuation are two long‐believed potential mechanisms for the reduced lattice thermal conductivity in high‐entropy ceramics (HECs). However, related studies remain unclear. Taking high‐entropy diborides (HEBs) as the prototype, the lattice‐distortion‐driven reduced lattice thermal conductivity in HECs is uncovered, whereas the influence of mass fluctuation is neglectable. Specifically, two groups of HEBs are designed by regulating the long‐believed mechanisms of lattice distortion and mass fluctuation based on machine‐learning‐potential‐based molecular dynamics simulations. The theoretical and experimental results show that lattice distortion plays a pivotal role in modulating the lattice thermal conductivity of HEBs, while the influence of mass fluctuation is neglectable. Further studies find that the aggravation of lattice distortion enables the reduction of the lattice thermal conductivity through the decreased phonon velocity and Debye temperature resulting from the simultaneously enhanced scattering of strain field fluctuation and bond strength fluctuation. In addition, lattice distortion is found to lower the electronic thermal conductivity by competing with vacancies. The research unravels the long‐standing mystery of the reduced lattice thermal conductivity in HECs and offers insightful guidance for developing HECs with ultra‐low thermal conductivities.

## Graphical abstract

Long‐believed potential mechanisms of lattice distortion and mass fluctuation for the reduced lattice thermal conductivity are explored by designing two groups of high‐entropy diborides based on machine‐learning‐potential‐based molecular dynamics simulations. The theoretical and experimental results uncover that lattice distortion plays a pivotal role in reducing the lattice thermal conductivity of HEBs, whereas the influence of mass fluctuation is neglectable.

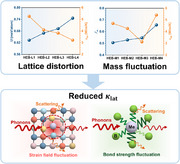

## 1 Introduction

High‐entropy ceramics (HECs), developed from the concept of high‐entropy alloys in 2015,^[^ ^1^ ^]^ are a novel class of inorganic materials characterized by their multi‐component composition, with each principal element constituting a significant atomic fraction (5–35%) of the total.^[^ ^2^, ^3^ ^]^ Based on this unique solid solution philosophy, which deviates from the traditional approach of relying on a single dominant element, different kinds of HECs, such as high‐entropy oxides,^[^ ^1^, ^4^, ^5^, ^6^ ^]^ high‐entropy diborides (HEBs),^[^ ^7^, ^8^, ^9^, ^10^, ^11^ ^]^ high‐entropy carbides,^[^ ^3^, ^12^, ^13^, ^14^, ^15^, ^16^, ^17^ ^]^ and high‐entropy nitrides,^[^ ^18^, ^19^, ^20^ ^]^ have hitherto been developed. These HECs have been shown to possess intriguing properties exceeding their individual counterparts, such as better thermal stability,^[^ ^1^, ^19^ ^]^ lower thermal conductivity,^[^ ^10^, ^19^ ^]^ enhanced mechanical properties,^[^ ^7^, ^11^, ^12^, ^13^ ^]^ improved corrosion and radiation resistance,^[^ ^7^, ^16^, ^21^ ^‐^ ^23^ ^]^ and superior electrochemical characteristics,^[^ ^5^, ^24^, ^25^ ^]^ owing to the four core effects of high‐entropy materials, including high‐entropy, lattice‐distortion, sluggish‐diffusion, and cocktail effects.^[^ ^2^ ^]^ Such exceptional properties have made HECs highly promising for a variety of structural and functional applications in aerospace, high‐end manufacturing, electronics, advanced electromagnetic protection, and catalysts.

**Figure 1:**
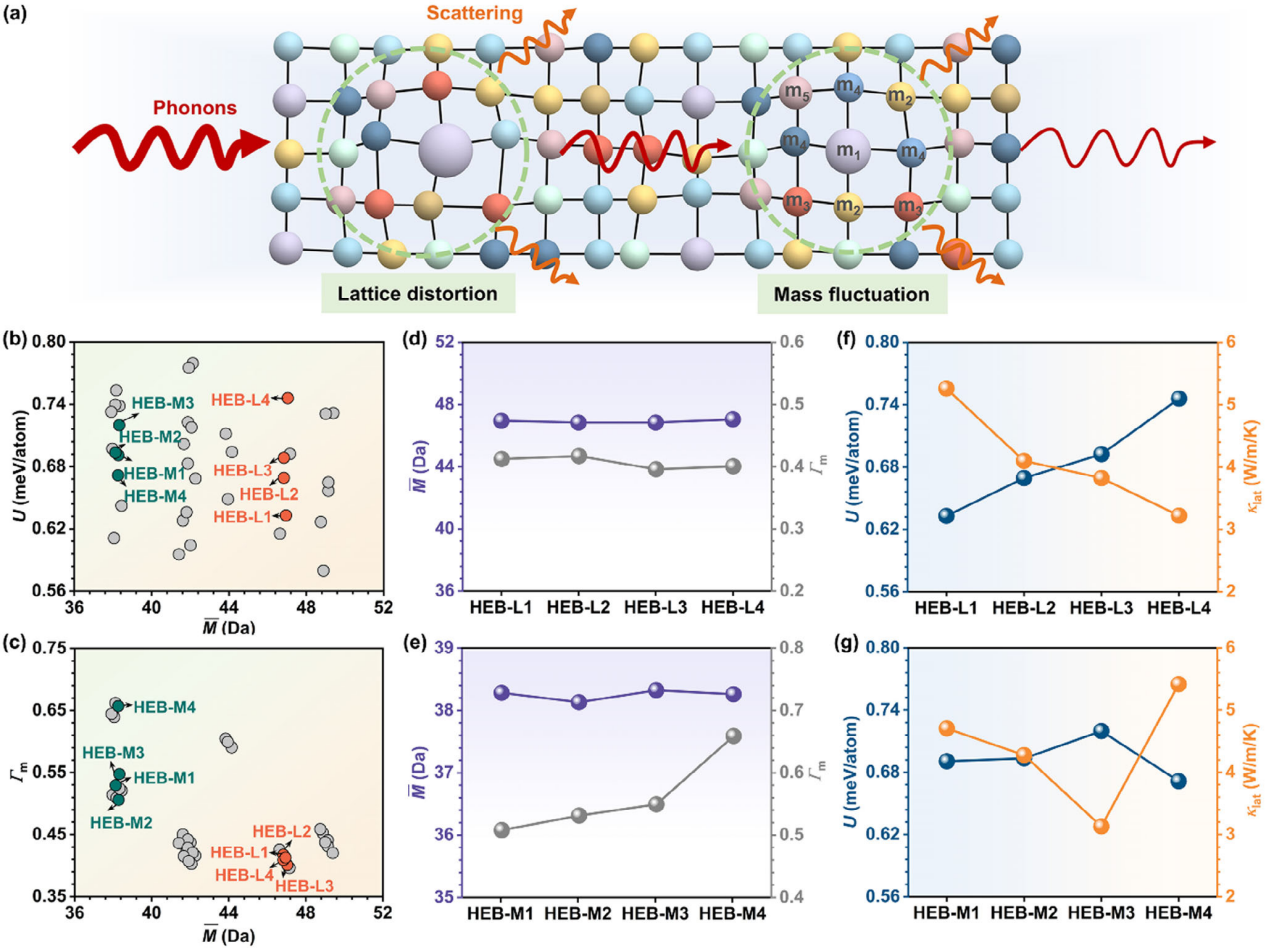
Design of two HEB groups by regulating lattice distortion and mass fluctuation. a) Schematic diagram of phonon scattering with lattice distortion and mass fluctuation. b) *U* versus $\bar{M}$ in HEBs. c) *Γ*_m_ versus $\bar{M}$ in HEBs. $\bar{M}$ and *Γ*_m_ of d) HEB‐L1−L4 and e) HEB‐M1−M4. *U* and *κ*_lat_ of f) HEB‐L1−L4 and g) HEB‐M1−M4.

Achieving ultralow thermal conductivity is the eternal pursuit of HECs to be used in aerospace, high‐end manufacturing, and electronics as insulating materials. Benefiting from the multi‐element solid solution features, HECs have shown the potential to break through the inherent thermal conductivity limits of conventional individual components,^[^ ^26^, ^27^, ^28^ ^]^ making them highly anticipated candidates to realize ultralow thermal conductivities. For instance, a considerably lower thermal conductivity than monolithic diborides was found in the newly synthesized (Hf, Ti, W, Zr)B_2_ HEB by Kavak et al.^[^ ^26^ ^]^ Similarly, Gild et al.^[^ ^27^ ^]^ reported decreased thermal conductivities in some quaternary and quinary high‐entropy fluorite oxides compared to their individual fluorite oxides. To understand the reduction in the thermal conductivity observed in these HECs, some potential mechanisms have been proposed, including mass fluctuations, lattice distortion, high‐entropy effects, and so on. Among them, mass fluctuations and lattice distortion, as inherent characteristics of multi‐element solid solution materials, are two long‐believed mechanisms that govern the thermal conductivity in HECs.^[^ ^28^, ^29^, ^30^, ^31^, ^32^, ^33^, ^34^ ^]^ As depicted in **Figure** **1**a, it is believed that server lattice distortion and enhanced mass fluctuation can cause great phonon scattering during the thermal transportation process, leading to a reduction of thermal conductivities.^[^ ^35^ ^]^ However, the consensus on their roles remains controversial. Some studies suggest that the combined effects of mass fluctuations and lattice distortion are responsible for the reduced thermal conductivity in HECs. For example, the decreased thermal conductivities in HEBs and high‐entropy rare‐earth titanates were believed to mainly arise from their enormous mass fluctuations and severe lattice distortion.^[^ ^29^, ^30^ ^]^ A prediction model based on both mass fluctuations and lattice distortion was also proposed to assess the thermal conductivity of medium‐entropy and high‐entropy rocksalt/pyrochlore oxides.^[^ ^31^ ^]^ On the contrary, some researchers thought that either mass fluctuation or lattice distortion alone led to a decrease in the thermal conductivity of HECs. Turcer et al.^[^ ^32^ ^]^ primarily attributed the low thermal conductivity of high‐entropy rare‐earth disilicates to the increased mass fluctuation with the incorporation of the additional cations, whereas Braun et al.^[^ ^33^ ^]^ asserted that lattice distortion resulted in the amorphous‐like thermal conductivity in their as‐synthesized high‐entropy rocksalt oxides. Unfortunately, these proposed mechanisms are all roughly discussed without solid evidence. Additionally, other potential mechanisms like high‐entropy effects have also been used to explain the decrease in the thermal conductivity of HECs.^[^ ^34^ ^]^ To sum up, research on the fundamental mechanisms that drive the reduction in the thermal conductivity of HECs is still in the early stages, significantly limiting the development of HECs with ultralow thermal conductivities for thermal insulating applications. Therefore, it is imperative to conduct a systematic investigation into the essence of the reduced thermal conductivity in HECs, advancing the design of desired thermal insulating materials with ultralow thermal conductivities.

The lattice thermal conductivity (*κ*_lat_) and the electronic thermal conductivity (*κ*_ele_) are the two principal contributors to the total thermal conductivity (*κ*_tot_) of HECs, where *κ*_lat_ is the most universal in all systems.^[^ ^36^, ^37^ ^]^ Here, we comprehensively investigate the underlying mechanism of the reduced *κ*_lat_ in HECs by focusing on HEBs—a typical member of HECs that have garnered significant research interest from the thermal insulation community because of their notably low thermal conductivities, ultrahigh melting points, and exceptional chemical inertness.^[^ ^7^, ^8^, ^9^, ^10^, ^11^, ^26^, ^29^ ^]^ Specifically, two sets of equimolar HEBs were first designed by regulating lattice distortion and mass fluctuation using machine‐learning‐potential‐based molecular dynamics (MD) simulations: one to assess the effect of lattice distortion on *κ*_lat_ by controlling mass fluctuations, and the other to evaluate the impact of mass fluctuations with minimal lattice distortion variation. On the basis of theoretical predictions and experimental measurements, lattice distortion is subsequently corroborated as essential in governing the *κ*_lat_ of HEBs, while mass fluctuation has a negligible influence on the *κ*_lat_. Further explorations were carried out to reveal the mechanism of the aggravated lattice distortion in reducing *κ*_lat_ of HEBs in depth. Our work elucidates the key mechanism behind the reduced *κ*_lat_ in HECs, providing valuable insight for the further design of HECs with ultralow thermal conductivities for thermal insulation applications.

## 2 Results and Discussion

To unveil the underlying mechanisms of the reduced *κ*_lat_ of HEBs, a single‐factor variable strategy was adopted in the design of HEBs to explore the influence of either lattice distortion or mass fluctuation systematically. Eight non‐magnetic transition metal elements (Me) from groups IIIB, IVB, VB, and VIB were first selected to design equimolar quaternary and quinary HEBs. These HEBs were then grouped with the similar average atomic mass ($\bar{M}$) of Me elements. In addition, the synthesis criteria for HEBs were also taken into account.^[^ ^38^ ^]^ As a result, a total of 37 potential quaternary and quinary HEBs were selected. For the sake of reliably evaluating lattice distortion, an accurate and transferable neuroevolution potential (NEP) for HEBs was first trained by our previous construction strategy based on the density functional theory (DFT) dataset of the unary and binary diborides.^[^ ^39^ ^]^ As shown in Figure S1a (Supporting Information), the NEP has been well‐trained, as evidenced by the effectiveness of the regularization (the increasing‐then‐decreasing trends of all regularization loss functions) and the convergence of energy, force, and virial losses. The accuracy and transferability of our trained NEP for HEBs were also confirmed with low testing root mean square errors (RMSEs) in energy (25.5 meV), force (196 meV Å^−1^), and virial (45.8 meV atom^−1^) for HEBs (see Figure S1b–d, Supporting Information). On the basis of this transferable NEP, the atomic strain energy (*U*) of HEBs can be calculated using MD simulations, which has been verified to be a reliable way to theoretically assess lattice distortion.^[^ ^11^ ^]^ Meanwhile, the mass fluctuation was quantified from the mass fluctuation scattering parameters (*Γ*_m_) to study its impacts on the *κ*_lat_ of HEBs.^[^ ^40^ ^]^ Besides lattice distortion and mass fluctuation, the average mass ($\bar{M}$) of Me atoms, which is proportional to *κ*_lat_ according to the classic Slack's model,^[^ ^41^ ^]^ was also taken into account (see Figure 1b,c), and their influence on the *κ*_lat_ should be eliminated. As a result, two groups of HEBs were selected: the first group contains four HEBs, including (Ti_1/5_Nb_1/5_Mo_1/5_Hf_1/5_Ta_1/5_)B_2_ (HEB‐L1), (Ti_1/5_Zr_1/5_Mo_1/5_Hf_1/5_Ta_1/5_)B_2_ (HEB‐L2), (V_1/5_Nb_1/5_Mo_1/5_Hf_1/5_Ta_1/5_)B_2_ (HEB‐L3), and (V_1/5_Zr_1/5_Mo_1/5_Hf_1/5_Ta_1/5_)B_2_ (HEB‐L4) that possess significantly increased *U* with almost constant $\bar{M}$ and *Γ*_m_ (see Figure 1d,f); the other group includes four HEBs, i.e., (Ti _1/5_V_1/5_Nb_1/5_Mo_1/5_Hf_1/5_)B_2_ (HEB‐M1), (Ti _1/5_V_1/5_Zr_1/5_Nb_1/5_Ta_1/5_)B_2_ (HEB‐M2), (Ti _1/5_V_1/5_Zr_1/5_Nb _1/5_W_1/5_)B_2_ (HEB‐M3), and (Ti _1/4_V_1/4_Nb_1/4_Ta_1/4_)B_2_ (HEB‐M4), which have noticeable changes in the *Γ*_m_ with minor variations in $\bar{M}$ and *U* (see Figure 1e,g). Detailed information on these two group HEBs and other considered HEBs is summarized in Table S1 (Supporting Information). Subsequently, the *κ*_lat_ of these two groups of HEBs was calculated by homogeneous nonequilibrium molecular dynamics (HNEMD) simulations (see Figures S2,S3, Supporting Information). It is notable that the *κ*_lat_ of HEB‐L1−L4 (Figure 1f) exhibits an inverse tendency with *U*, indicating that intensified lattice distortion is beneficial to reducing *κ*_lat_ of HEBs. In contrast, the calculated *κ*_lat_ in HEB‐M1−M4 fluctuates as the *Γ*_m_ monotonically increases (Figure 1e,g), suggesting a neglectable impact of mass fluctuation on *κ*_lat_. Interestingly, there is still a strong correlation between *κ*_lat_ and *U* in HEB‐M1−M4 (see Figure 1g), further confirming the critical role of lattice distortion in tuning the *κ*_lat_. Therefore, it can be inferred that lattice distortion is the pivotal factor responsible for reducing *κ*_lat_ in HEBs, while the influence of mass fluctuation is marginal.

**Figure 2:**
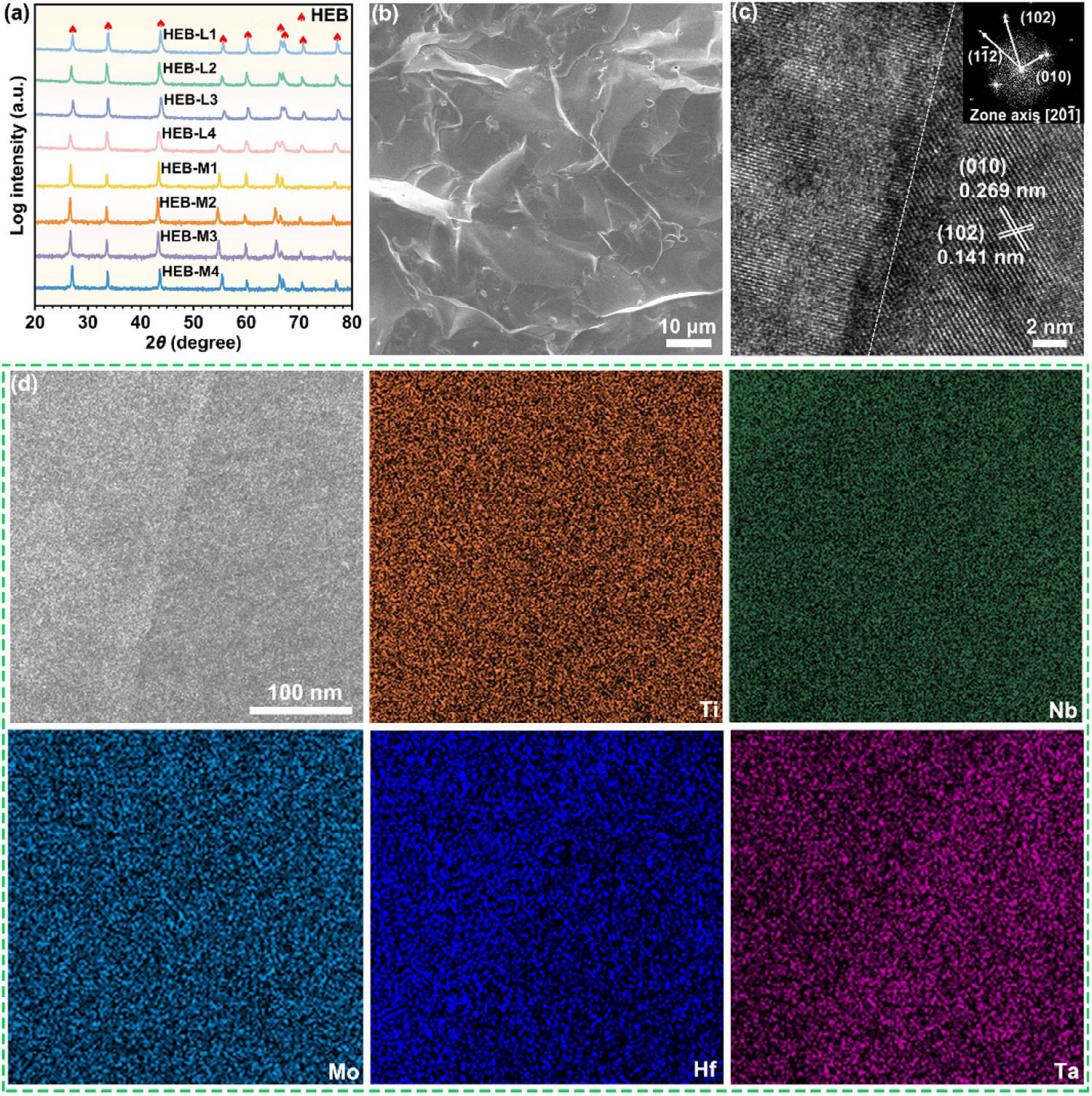
Crystal structure and morphology analysis of the as‐fabricated HEB‐L1−L4 samples. a) XRD patterns of the as‐fabricated HEB‐L1−L4 and HEB‐M1−M4 samples. b) Fractured surface SEM image of the as‐fabricated HEB‐L1 samples. c) HRTEM image and corresponding FFT pattern of the as‐fabricated HEB‐L1 samples. d) STEM image and the corresponding EDS compositional maps of the as‐fabricated HEB‐L1 samples.

To experimentally verify our MD predictions and provide a further in‐depth understanding, the HEB‐L1−L4 and HEB‐M1−M4 samples were first fabricated by a two‐step technique involving ultrafast high–temperature sintering (UHTS) and spark plasma sintering (SPS). The X‐ray diffraction (XRD) patterns (**Figure** **2**a), along with corresponding Rietveld refinements with low fitting parameters (*R*_wp_ and *R*_p_) (Figure S4, Supporting Information) and well‐refined unit cell parameters with DFT calculation results (**Table** **1**), verify that the single‐phase HEBs with hexagonal structures (space group *P*6*/mmm*) have been successfully fabricated as designed. Besides the crystal structures, the relative densities and average grain sizes (determined by fitting a Gaussian function of the grain size distribution in Figure S5, Supporting Information) in all the as‐fabricated HEB samples are measured. Taking HEB‐L1−L4 as an examples, Figure S6 (Supporting Information) demonstrates that their relative densities and average grain sizes are comparable with tiny variations. Moreover, the scanning electron microscope (SEM) image of the representative HEB‐L1 samples (see Figure 2b) demonstrates that they tend to fracture in an intergranular mode, indicating strong interfacial bonding between grain boundaries. The high‐resolution transmission electron microscopy (HRTEM) image and the corresponding fast Fourier transform (FFT) pattern (see Figure 2c) further exhibit that the grain boundaries of the representative HEB‐L1 samples are distinct and clear without any impurities, confirming their high‐quality interfaces that feature strong interfacial bonding. Meanwhile, the periodic lattice structure shows two sets of fringes, i.e., interplanar spacings of 0.269 and 0.141 nm (corresponding to {010} and {102} planes of metal diborides, respectively), yielding calculated lattice parameters *a* and *c* of 3.084 and 3.269 Å, respectively. These resultant lattice parameters are in line with XRD refinements (3.082 and 3.319 Å) and DFT calculations (3.086 and 3.249 Å), further verifying the successful fabrication of single‐phase HEB‐L1 samples. In addition, the elemental homogeneity of the as‐fabricated HEB‐L1−L4 samples is examined. The SEM images and corresponding energy dispersive spectroscopy (EDS) maps in Figure S7 (Supporting Information) indicate that Me elements with equimolar ratios (see Table S2, Supporting Information) are all homogeneously distributed at the micron scale without any segregation or aggregation. Such good compositional uniformity is also confirmed at the nanoscale, as evidenced by the scanning transmission electron microscopy (STEM) images and the corresponding EDS maps (see Figure 2d; Figure S8, Supporting Information), as well as the measured atomic percentages (see Table S3, Supporting Information). Notably, the Me elemental distribution of grain boundaries is also uniform with no segregation or aggregation, further demonstrating good interfacial bonding. Based on these experimental results, it can be concluded that all the as‐designed HEB samples have been well fabricated with comparable structural and compositional characteristics, and therefore, the effects of relative densities, grain sizes, grain boundaries, and elemental distributions on the thermal conductivities of our as‐fabricated HEB samples can be ruled out.

**Table 1:** Lattice parameters (*a* and *c*) of the as‐fabricated HEB‐L1−L4 and HEB‐M1−M4 samples from XRD and DFT calculations.

Samples	*a*_XRD_ [Å]	*c*_XRD_ [Å]	*a*_cal_ [Å]	*c*_cal_ [Å]
HEB‐L1	3.082	3.319	3.086	3.329
HEB‐L2	3.079	3.316	3.101	3.367
HEB‐L3	3.074	3.302	3.076	3.312
HEB‐L4	3.087	3.354	3.091	3.349
HEB‐M1	3.093	3.359	3.068	3.274
HEB‐M2	3.102	3.376	3.083	3.303
HEB‐M3	3.096	3.370	3.074	3.286
HEB‐M4	3.087	3.331	3.061	3.237

**Figure 3:**
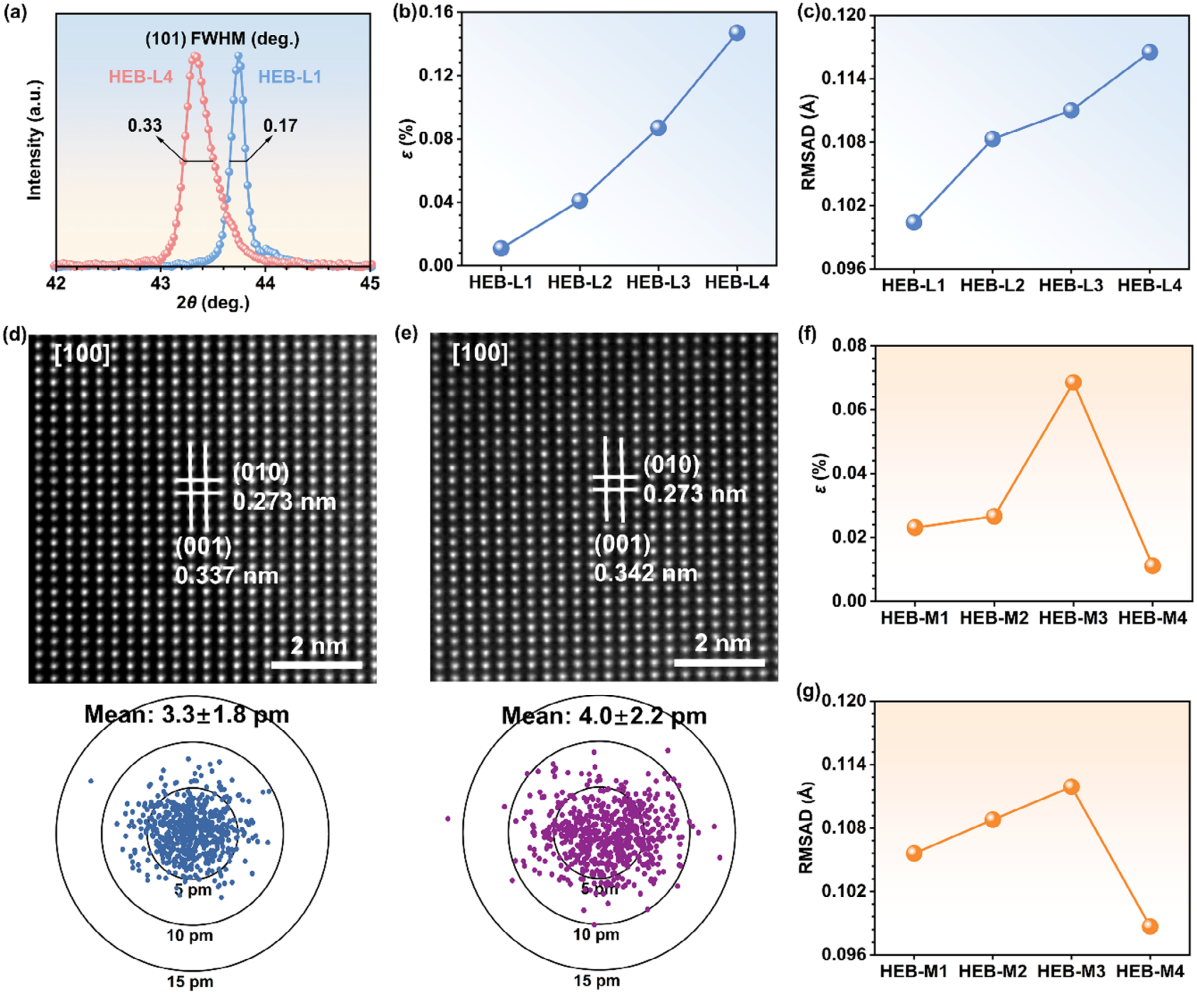
Lattice distortion from XRD and HAADF‐STEM images. a) Comparison of FWHMs of selected characteristic peaks between the as‐fabricated HEB‐L1 and HEB‐L4 samples. b) *ε* of the as‐fabricated HEB‐L1−L4 samples from XRD. c) RMSAD of HEB‐L1−L4. HAADF‐STEM images along the [100] zone axis and the corresponding mean atomic displacements of the as‐fabricated d) HEB‐L1 and e) HEB‐L4 samples. f) *ε* of the as‐fabricated HEB‐M1−M4 samples from XRD. g) RMSAD of HEB‐M1−M4.

Lattice distortion of our as‐fabricated HEB samples is further experimentally measured through XRD and the high‐angle annular dark field scanning transmission electron microscopy (HAADF‐STEM). Taking the HEB‐L1 and HEB‐L4 as an example, the enlarged XRD patterns for the (101) characteristic peaks (see **Figure** **3**a) demonstrate that the full width at half maximum (FWHM) of the as‐fabricated HEB‐L4 samples is apparently larger than that of the as‐fabricated HEB‐L1 samples. Given such significant differences in FWHM, the lattice strain (*ε*) of all the as‐fabricated HEB samples have been fitted by the Williamson‐Hall analysis.^[^ ^42^ ^]^ As illustrated in Figure S9 (Supporting Information) and Figure 3b, the continuously increased *ε* from HEB‐L1 to HEB‐L4 indicates the enlarged lattice distortion in the as‐fabricated HEB‐L1−L4 samples at the macroscopic scale, which is well consistent with our design. Such a variation in lattice distortion can be further validated by computing the root mean squared atomic displacement (RMSAD) based on MD simulations. As displayed in Figure 3c, the RMSAD climbs monotonically from HEB‐L1 to HEB‐L4, confirming the intensified lattice distortion from HEB‐L1 to HEB‐L4. Furthermore, the HAADF‐STEM imaging was employed to explicitly evaluate lattice distortion at the atomic scale, where the atomic displacement away from ideal sites (i.e., lattice distortion) of the as‐fabricated samples can be directly measured. As shown in Figure 3d,e, the as‐fabricated HEB‐L4 samples exhibit a more significant mean atomic displacement than that of the as‐fabricated HEB‐L1 samples (4.0 ± 2.2 pm vs 3.3 ± 1.8 pm) along the [100] orientation. This observation is in good agreement with the calculations of the RMSAD, providing solid evidence to support the enhanced lattice distortion in the as‐fabricated HEB‐L1−L4 samples. Additionally, the *ε* and the RMSAD of the as‐fabricated HEB‐M1−M4 samples are presented in Figure 3f,g. The fluctuated tendencies of both *ε* and RMSAD show fluctuated lattice distortion from the as‐fabricated HEB‐M1 to HEB‐M4 samples, which is also consistent with the computed *U* in Figure 1g. Therefore, lattice distortion of the as‐fabricated HEB samples is determined to align well with our initial design in both groups.

**Figure 4:**
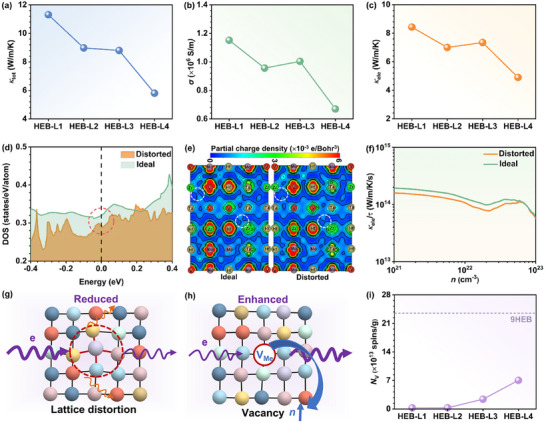
Measured thermal conductivity and mechanism of the lattice‐distortion‐driven reduced *κ*_ele_. a) *κ*_tot_ of the as‐fabricated HEB‐L1−L4 samples. b) Conductivity of the as‐fabricated HEB‐L1−L4 samples. c) *κ*_ele_ of the as‐fabricated HEB‐L1−L4 samples. d) DOS of the ideal and distorted structure of HEB‐L4. Fermi level is set to 0 eV. e) Partial charge density of the ideal and distorted structure of HEB‐L4. f) *κ*_ele_/*τ* of the ideal and distorted structure of HEB‐L4 at 300 K as a function of *n*. g) Schematic diagram of lattice distortion on reducing *κ*_ele_. h) Schematic diagram of vacancy on enhancing *κ*_ele_. i) *N*_v_ of the as‐fabricated HEB‐L1−L4 samples and the reported 9HEB.^[^ ^43^ ^]^

Further studies on the thermal conductivities of the as‐fabricated HEB samples were conducted. As illustrated in **Figure** **4**a, the *κ*_tot_ decreases monotonically from the as‐fabricated HEB‐L1 samples to HEB‐L4 samples, showing an opposite trend with lattice distortion. To distinguish the electronic and lattice contributions of the thermal conductivities, the electrical conductivity (*σ*) of the as‐fabricated HEB‐L1−L4 samples was measured. As shown in Figure 4b, an overall declining trend of the σ can be observed from the as‐fabricated HEB‐L1 to HEB‐L4 samples. Such a reduced σ can lead to a corresponding decrease of *κ*_ele_ (*κ*_ele_∝*σ*, more calculation details are described in the Experimental Section/Methods) from the as‐fabricated HEB‐L1 to HEB‐L4 samples (see Figure 4f), implying that the critical role of aggravation of lattice distortion in reducing *κ*_ele_. To further elucidate the impact of lattice distortion on the *κ*_ele_ reduction, HEB‐L4 was taken as the model, and its density of states (DOS) with and without lattice distortion (i.e., distorted and ideal *P*6*/mmm* structure, respectively) were first explored. As shown in Figure 4d, DOS at the Fermi level of the distorted HEB‐L4 is calculated to be lower than that of the ideal one, indicating a reduction of metallicity due to lattice distortion and, thereby, a reduced *κ*_ele_. Moreover, a comparison of the partial charge densities of the distorted and ideal HEB‐L4 was carried out. It is obvious that the distorted HEB‐L4 structure possesses fewer conductive channels (emphasized by the white circle in Figure 4e) than the undistorted one, suggesting the poorer conductance of HEB‐L4 with lattice distortion. Additionally, the electrical transportation properties at 300 K were calculated using the Boltzmann transport equation. As demonstrated in Figure 4f, given the same electron carrier concentration (*n*), the distorted HEB‐L4 demonstrates a consistently lower normalized *κ*_ele_ over the relaxation time (*τ*) compared to the undistorted one, which further implies its reduced *κ*_ele_. Based on the aforementioned analyses, it can be deduced that lattice distortion contributes to the reduction of the *κ*_ele_ (see Figure 4g). However, it is important to note that our previous study also reported an improved σ with aggravated lattice distortion in the 9‐cation HEB.^[^ ^43^ ^]^ Such a reverse tendency in σ should be associated with the competition between lattice distortion and lattice distortion‐induced vacancy concentration (*N*_v_) in HEBs. In other words, a dual effect of lattice distortion on σ can be inferred: on the one hand, increasing lattice distortion can directly lead to a lowered σ, as well as a reduced *κ*_ele_; on the other hand, severe lattice distortion will induce the formation of more metal vacancies in HEBs, resulting in higher carrier concentrations (*n*) and *σ*, as well as an increased *κ*_ele_ (see Figure 4h). This speculation can be verified by comparing the *N*_v_ of our as‐fabricated HEB‐L1−L4 samples with the reported 9‐cation HEB in Figure 4i, where the largest *N*_v_ of the as‐fabricated HEB‐L1−L4 samples is an order of magnitude smaller than that of the reported 9‐cation HEB. Thus, lattice distortion undergoes a rivalry with the vacancy, ultimately leading to a decrease in the *κ*_ele_ of HEBs.

**Figure 5:**
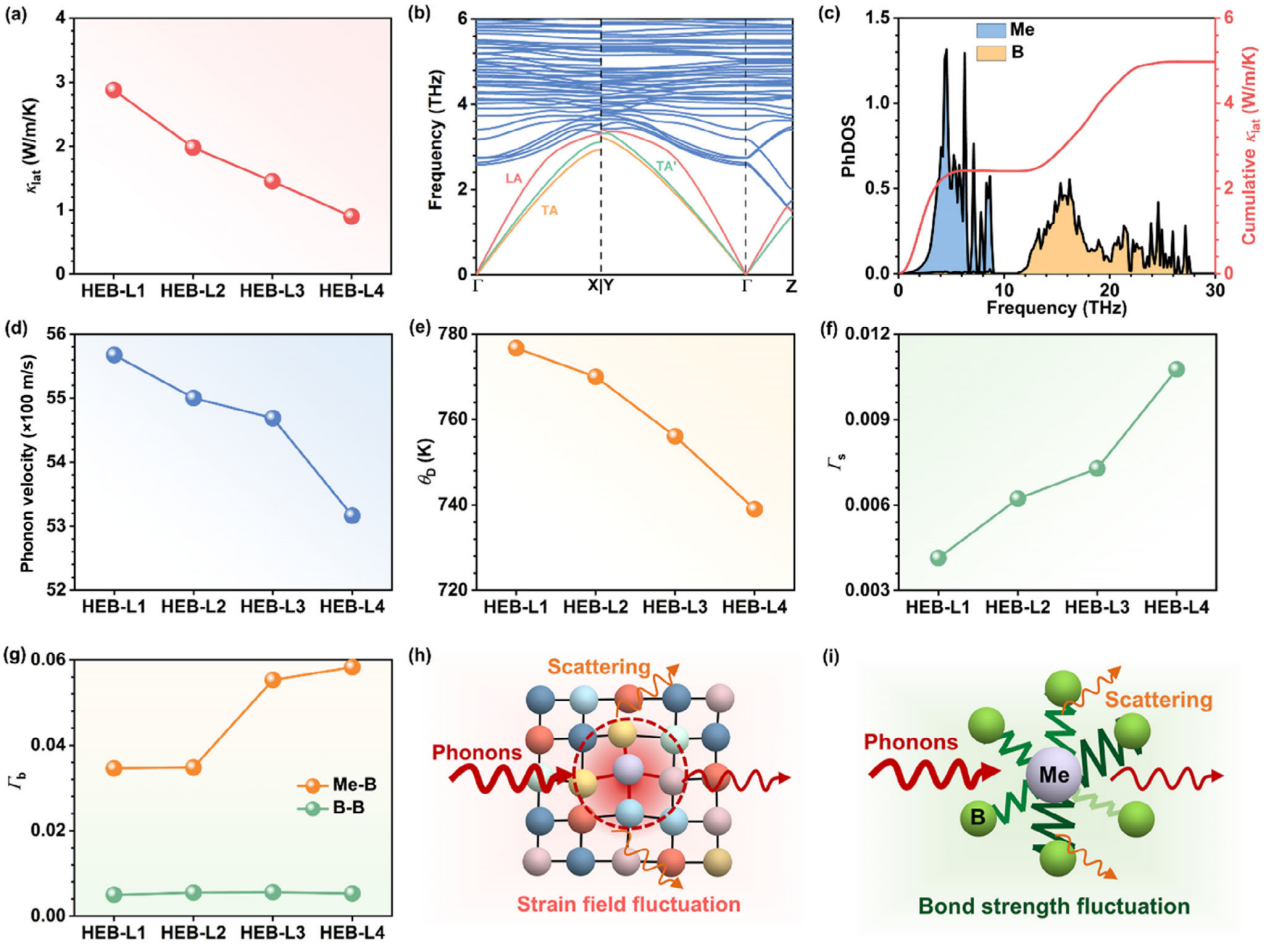
Mechanism of the lattice‐distortion‐driven reduced *κ*_lat_. a) *κ*_lat_ of the as‐fabricated HEB‐L1−L4 samples. b) Phonon dispersions of HEB‐L1. TA, yellow color; TA’, green color; LA, red color. c) PhDOS and cumulative *κ*_lat_ of HEB‐L1. d) Phonon velocity of HEB‐L1−L4. e) *θ*_D_ of HEB‐L1−L4. f) *Γ*_s_ of HEB‐L1−L4. g) *Γ*_b_ of HEB‐L1−L4. h) Schematic diagram of strain field fluctuation in reducing *κ*_lat_. i) Schematic diagram of bond strength fluctuation in reducing *κ*_lat_.

With the *κ*_tot_ and *κ*_ele_, the *κ*_lat_ of the as‐fabricated HEB‐L1−L4 samples can be obtained. As shown in **Figure** **5**a, it is found that the *κ*_lat_ exhibits a monotonically declined trend from the as‐fabricated HEB‐L1 samples to HEB‐L4 samples. This trend aligns with the calculated *κ*_lat_ in Figure 1f, further confirming the accuracy of our MD predictions. The same conclusion can be drawn from the measured *κ*_lat_ of the as‐fabricated HEB‐M1−M4 samples in Figure S10 (Supporting Information), which possess the same trend as our calculations in Figure 1g. Hence, the pivotal role of lattice distortion as well as the neglected mass fluctuation in the reduction of *κ*_lat_ has been experimentally and theoretically verified. To gain a deeper understanding of lattice distortion on the *κ*_lat_ reduction, phonon dispersions of HEB‐L1−L4 were studied in Figure 5b and Figure S11a–c (Supporting Information). It is obvious that there are no imaginary frequencies in the phonon dispersions of HEB‐L1−L4, indicating that they are all lattice dynamically stable. Meanwhile, the phonon DOS (PhDOS) with a q‐mesh of 10 × 10 × 10 and cumulative *κ*_lat_ are shown in Figure 5c and Figure S11d–f (Supporting Information). Notably, both the Me and boron (B) atoms are determined to have a comparable contribution to the *κ*_lat_ in HEB‐L1−L4. Furthermore, the average phonon velocity and the Debye temperature (*θ*_D_) were investigated since both of them are theoretically proportional to the *κ*_lat_.^[^ ^44^ ^]^ As presented in Figure 5d, the average phonon velocity of HEB‐L1−L4 was computed from the slopes of the longitudinal acoustic (LA) and transverse acoustic (TA and TA’) phonon branches around the Γ point along three principal Brillouin zone paths in Figure 5b. The detailed phonon velocities of LA, TA, and TA’ acoustic branches are listed in Table S4 (Supporting Information), which is consistent with the ones evaluated from the elastic tensors. It is clear that the average phonon velocity decreases gradually from HEB‐L1 to HEB‐L4, demonstrating the reduced *κ*_lat_ from HEB‐L1 to HEB‐L4. In addition, the *θ*_D_ is also calculated to decrease from HEB‐L1 to HEB‐L4 (see Figure 5e), implying a reduction of the *κ*_lat_ in HEB‐L1−L4. To clarify the origin of the reduction in the phonon velocity and *θ*_D_ caused by lattice distortion, phonon‐disorder scattering mechanisms were further analyzed. The strain field fluctuation scattering parameter (*Γ*_s_) is plotted in Figure 5f. It can be seen that the server lattice distortion can significantly enhance *Γ*_s_, bringing an increase in phonon scattering and the reduction of the *κ*_lat_. This result can also be identified by the observation of the enhanced atomic volumetric and shear strain fluctuation in HEB‐L1−L4 (see Figure S12, Supporting Information). Additionally, the bond strength of Me‐B and B‐B was calculated from the integrated‐crystal orbital Hamilton population (‐ICOHP) to evaluate the scattering from bond strength fluctuation (*Γ*_b_). Figure 5g shows the *Γ*_b_ of HEB‐L1−L4, where the *Γ*_b_ of Me‐B becomes larger from HEB‐L1 to HEB‐L4 while the *Γ*_b_ of B‐B remains unchanged. These phenomena suggest that the aggravation of lattice distortion in HEB‐L1−L4 enables the enhanced scattering of *Γ*_b_ in Me─B bonds, which in turn contributes to the reduction of the *κ*_lat_. This finding also aligns with the comparable contribution of both Me and B atoms for the *κ*_lat_ in Figure 5c. Therefore, lattice distortion is unraveled to drive the reduction of both phonon velocities and *θ*_D_ by simultaneously increasing the scattering from *Γ*_s_ and *Γ*_b_ (Figure 5h,i), eventually resulting in the reduced *κ*_lat_.

## 3 Conclusion

In summary, the underlying mechanism of lattice‐distortion‐driven reduced *κ*_lat_ in HECs has been unraveled by investigating the properties of HEBs. To be specific, two groups of HEBs have been designed to control either lattice distortion or mass fluctuation using machine‐learning‐potential‐based MD simulations. With a combination of MD predictions and experimental verifications, lattice distortion has been found to play a pivotal role in governing the *κ*_lat_ of HEBs, while the impact of mass fluctuation has been determined to be insignificant. Further studies have revealed that an aggravated lattice distortion could result in a significantly decreased *κ*_lat_ through the reduction of both phonon velocities and *θ*_D_ by increasing the scattering of both *Γ*_s_ and *Γ*_b_. In addition, lattice distortion has the potential to lower *κ*_ele_ by competing with vacancies. Our results provide a deep understanding of lattice‐distortion‐driven reduced *κ*_lat_ in HECs, accelerating the design of new thermal insulating HECs with ultra‐low thermal conductivities.

## 4 Experimental Section

### NEP Constructions and MD Simulations

DFT static calculations on 3613 configurations were performed to build the training dataset (3088, unary and binary diborides) and the testing dataset (525, from equimolar quaternary HEBs to equimolar octonary HEBs) for the NEP training within the Graphics Processing Units Molecular Dynamics (GPUMD) code.^[^ ^45^ ^]^ The detailed data collection strategy and training hyperparameters can be found in the previous works.^[^ ^39^ ^]^ The Large Scale Atomic/Molecular Massively Parallel Simulator (LAMMPS) package was applied to perform MD simulations interfaced with the NEP code.^[^ ^46^ ^]^ All the simulated HEB models were built by randomizing the metal elements in a 20 × 20 × 20 TiB_2_ orthorhombic supercell (a total of 48000 atoms). All the models were relaxed at 300 K for 10 ps under the NPT ensemble and equilibrated for 10 ps in the NVT ensemble. A time step of 1 fs and periodic boundary conditions used in all directions were set throughout all simulations. The structure, the volume (*V^i^*), the atomic stress tensor ($\varepsilon_{ab}^{i}$, *a* and *b* are the notation of *x*, *y*, and *z*), and the atomic stress tensor ($\sigma_{ab}^{i}$) of the i‐th atom were collected from the last 2 ps to evaluate the *U* as lattice distortion:^[^ ^11^ ^]^

(1) $$\mathrm{Volumetric}\;\mathrm{strain}\;=\frac{\varepsilon_{xx}^{i}+\varepsilon_{yy}^{i}+\varepsilon_{zz}^{i}}{3}\;$$

(2)

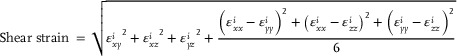

(3) $$\begin{array}{c} U=\frac{\;\sum_{i}^{N}\frac{1}{2}V^{i}\left(\sigma_{xx}^{i}\varepsilon_{xx}^{i}+\sigma_{yy}^{i}\varepsilon_{yy}^{i}+\sigma_{zz}^{i}\varepsilon_{zz}^{i}+2\sigma_{xy}^{i}\varepsilon_{xy}^{i}+2\sigma_{yz}^{i}\varepsilon_{yz}^{i}+2\sigma_{xz}^{i}\varepsilon_{xz}^{i}\right)}{N}\; \end{array}$$

where *N* is the number of atoms in the supercells. In addition, the RMSAD can also be calculated to quantify the lattice distortion from the aspects of atomic‐position deviation as follows:^[^ ^47^ ^]^

(4) $$\mathrm{RMSAD}\;=\frac{\sum_{j}\sqrt{\frac{\sum_{i}^{n}{\left(x_{i}^{\mathrm{relaxed}}-x_{i}^{\mathrm{ideal}}\right)}^{2}}{n}}}{2000}\;$$

where $x_{i}^{\mathrm{ideal}}$ is the ideal lattice site of the *i*‐the atom, $x_{i}^{\mathrm{relaxed}}$ is the atomic position after relaxation, and *j* is the *j*‐th step in the last 2 ps. The relaxed and equilibrated models were first equilibrated for 1 ps and then collected the following 2 ns heat current data in the NVT ensemble at 300 K to predict *κ*_lat_ through the HNEMD method in the GPUMD package with a timestep of 1 fs and periodic boundary conditions.^[^ ^45^, ^48^ ^]^ The driving force was chosen as *F_e_* = 0.0001 Å^−1^ (see convergence tests in Figure S13a, Supporting Information), and three different transport directions with four independent simulations for each model (the finite size effects of the model were checked in Figure S13b, Supporting Information) were performed to calculate the averaged *κ*_lat_ in the range between 1 and 2 ns. A total correlation step of 200, a maximum angular frequency of 400 THz, and a sample interval of 5 were used for three different transport directions to perform *κ*_lat_(*ω*) calculations. The average mass and mass fluctuations for a compound A*_x_* B*_y_*, which has two sublattices, were defined as follows:^[^ ^40^ ^]^

(5) $$\bar{M}=\frac{x{\bar{M}}_{x}+y{\bar{M}}_{y}}{x+y}$$

(6) $${\bar{M}}_{x}=\sum_{i}j_{i}m_{i}\;$$

(7) $${\bar{M}}_{y}=\sum_{i}k_{i}m_{i}\;$$

(8) $$\begin{array}{ccc} \Gamma_{m} & = & \frac{x}{x+y}\;{\left(\frac{{\bar{M}}_{x}}{\bar{M}}\right)}^{2}\left(\sum_{i}j_{i}{\left(\frac{m_{i}-{\bar{M}}_{x}}{{\bar{M}}_{x}}\right)}^{2}\right)\\ & & +\frac{y}{x+y}{\left(\frac{{\bar{M}}_{y}}{\bar{M}}\right)}^{2}\left(\sum_{i}k_{i}{\left(\frac{m_{i}-{\bar{M}}_{y}}{{\bar{M}}_{y}}\right)}^{2}\right) \end{array}$$

where $\bar{M}$ is the average mass, ${\bar{M}}_{x}$ and ${\bar{M}}_{y}$ are the average mass at sites *x* and *y*, *j_i_* and *k_i_* are the fractional occupations at sites *x* and *y*, and *m_i_* is the *i*‐th mass at sites *x* and *y*, respectively. In order to obtain *θ*_D_, temperature‐dependent elastic tensors were computed at 300 K to evaluate the bulk modulus (*B*) and the shear modulus (*G*) through the Voigt–Reuss–Hill method,^[^ ^49^ ^]^ by taking Born term into account within 10 ps and the magnitude of strain was set to 10^−6^. *θ*_D_ and the average speed of sound in the elastic mechanics (*v*_s_) can be calculated as follows:^[^ ^50^ ^]^

(9) $$\theta_{D}=\frac{hv_{s}}{k_{B}}\;{\left(\frac{3}{4\pi }\frac{N_{p}}{V_{p}}\right)}^{\frac{1}{3}}$$

(10) $$v_{s}={\left(\frac{1}{3}\left(\frac{1}{v_{l}^{3}}+\frac{2}{v_{t}^{3}}\right)\right)}^{-\frac{1}{3}}\;$$

(11) $$v_{l}=\sqrt{\frac{B+\frac{4}{3}G}{\rho }}\;$$

(12) $$v_{t}=\sqrt{\frac{G}{\rho }}\;$$

where *V*_p_ is the volume of the primitive cell, *N*_p_ is the number of atoms in the primitive cell, *ρ* is the density of the material, *h* is the Planck constant, and *k*_B_ is the Boltzmann constant. For phonon calculations based on small displacement methods,^[^ ^51^ ^]^ 2 × 2 × 5 supercells of HEBs were used to obtain the 2nd‐order interatomic force constants from 20 ps MD simulations in the NVT ensemble at 300 K.^[^ ^52^, ^53^ ^]^ The average phonon group velocity was obtained by averaging the slopes of LA, TA, and TA′ phonon branches.

### DFT Calculations

All DFT calculations were performed using the Vienna ab initio simulation package (VASP).^[^ ^54^ ^]^ The projector augmented wave (PAW) combined with the Perdew‐Burke‐Ernzerhof (PBE) under the generalized gradient approximation (GGA) was applied to approximate the electronic exchange and correlation function.^[^ ^55^, ^56^ ^]^ The special quasi‐random structure (SQS) approach performed in the Alloy Theoretic Automated Toolkit (ATAT) was applied to generate the disordered occupations of the Me atoms in HEB supercells based on TiB_2_ conventional cell (4 × 4 × 5 for all HEB supercells).^[^ ^57^, ^58^ ^]^ The plane‐wave cutoff energy was set to 420 eV. The Brillouin zone was sampled employing the Γ‐centered method with a separation of 0.3 Å^−1^.^[^ ^59^ ^]^ The energy convergence criterion of the electronic self‐consistency cycle was set to 10^−5^ eV. DOS was calculated using a denser 6 × 6 × 4 k‐point grid. Based on the DOS calculations, the electrical transport properties of HEB‐L4 ideal and distorted structures were further computed using the Boltzmann transport theory as performed in the BoltzTraP code.^[^ ^60^ ^]^ ‐ICOHP was calculated to analyze the strength fluctuation scattering of Me─B and B─B bonds in HEBs.^[^ ^61^ ^]^ The *Γ*_s_ was estimated as follows:^[^ ^40^ ^]^

(13) $$\begin{array}{ccc} \Gamma_{s} & = & \frac{x}{x+y}\;{\left(\frac{{\bar{M}}_{x}}{\bar{M}}\right)}^{2}\left(\sum_{i}j_{i}{\left(\frac{r_{i}-{\bar{r}}_{x}}{{\bar{r}}_{x}}\right)}^{2}\right)\\ & & +\frac{y}{x+y}{\left(\frac{{\bar{M}}_{y}}{\bar{M}}\right)}^{2}\left(\sum_{i}k_{i}{\left(\frac{r_{i}-{\bar{r}}_{y}}{{\bar{r}}_{y}}\right)}^{2}\right) \end{array}$$

where *r_i_* is the *i*‐th atom radii and ${\bar{r}}_{x}$ and ${\bar{r}}_{y}$ are the average radii at sites *x* and *y*. The strength fluctuation scattering of Me─B and B─B bonds in HEBs can also be defined as the following equation:

(14) $$\Gamma_{b}=\sum_{i}j_{i}{\left(\frac{b_{i}-\bar{b}}{\bar{b}}\right)}^{2}\;$$

where *b_i_* is the *i*‐th bond strength obtained from ‐ICOHP, and $\bar{b}$ is the average bond strength.

### Sample Synthesis

HEB‐L1−L4 and HEB‐M1−M4 samples were fabricated using two‐step UHTS and SPS methods. Commercial metal oxides, including HfO_2_, Ta_2_O_5_, Nb_2_O_5_, ZrO_2_, TiO_2_, V_2_O_5_, MoO_3_, WO_3_ (purity: 99.9%, particle size: 1–3 µm, McLean Biochemical Technology Co., Ltd., China), and amorphous B_4_C powders (purity: 99%, particle size: 10–20 µm, McLeanBiochemical Technology Co., Ltd., China), were prepared as raw materials. First, the metal oxide powders with equal molar ratios of metal elements and 40%‐excess B_4_C powders were weighed based on the different compositions of as‐designed HEB‐L1−L4 and HEB‐M1−M4. Subsequently, the powders were wet‐ball‐milled for 24 hours in ethanol using high‐purity ZrO_2_ balls and then dried by applying a rotary evaporator to obtain uniform mixtures. Afterward, the mixtures were compacted into 16 mm × 16 mm × 3 mm pellets under a pressure of 6 MPa and held for 4 min, followed by being wrapped in graphite paper and placed in graphite felt (Av Carb Felt G650, Fuel Cell Store, USA) with a size of 100 mm ×20 mm ×5 mm. The pellets were rapidly heated to 2273 K for 1 min in an argon atmosphere, followed by naturally cooling to room temperature to obtain the desired HEB powders. Finally, the as‐synthesized HEB powders were poured into a graphite mold with a diameter of 15 mm and sintered at 2273 K for 15 min under a pressure of 45 MPa using the SPS apparatus. The as‐fabricated HEB samples were polished with 1200‐grid SiC paper and were cut into the bulks with a size of 10 mm× 10 mm× 2 mm for the subsequent thermal conductivity tests.

### Materials Characterization

The phase composition of the as‐fabricated samples was analyzed by XRD (X′pert PRO; PANalytical, Netherlands). The refinement was performed by using a general structure analysis system (GSAS) software. The thermal diffusivity (*h*) was measured at room temperature under an argon flow by a laser‐flash apparatus (Netzsch LFA 427, Netzsch, Selb, Germany) to obtain thermal conductivity. $\kappa_{\mathrm{tot}}^{0}$ was evaluated as follows:^[^ ^62^ ^]^

(15) $$\kappa_{\mathrm{tot}}^{0}=h\cdot \rho_{A}\cdot C_{p}$$

where *C_p_* is the specific heat capacity estimated based on the Dulong‐Petit law and *ρ*_A_ is the density of the samples measured by the Archimedes drainage method. The *κ*_tot_ of a fully dense specimen is calculated according to the following equation:^[^ ^63^ ^]^

(16) $$\kappa_{\mathrm{tot}}=\frac{\kappa_{\mathrm{tot}}^{0}}{1-\frac{4}{3}\varphi }\;$$

where *φ* is the porosity. In addition, the σ of samples at room temperature was measured on a commercial apparatus (CTA‐3, Cryoall, Beijing, China) under a helium atmosphere and the corresponding *κ*_ele_ was computed according to Wiedemann‐Franz law:^[^ ^64^ ^]^

(17) $$\kappa_{\mathrm{ele}}=L\cdot \sigma \cdot T$$

where *L* = 2.44 × 10^−8^ W Ω K^−2^ is the Lorenz number, and *T* is the absolute temperature. The *κ*_lat_ was thereby obtained:

(18) $$\kappa_{\mathrm{lat}}=\kappa_{\mathrm{tot}}\;-\kappa_{\mathrm{ele}}$$

In addition, the vacancy defect was tested at room temperature by electron paramagnetic resonance (EPR, A300, Bruker, Billerica, USA). The microstructure and compositional uniformity of the as‐fabricated samples were examined on both micrometer and nanometer scales using SEM (Supra‐55, Zeiss, Germany) with EDS and TEM (Talos F200x, Thermo Fisher Scientific, USA), respectively. To prepare the samples for TEM analysis, several lamellae, with dimensions of 5 µm × 8 µm × 0.1 µm, were cut from the surface of the samples using a focused ion beam (FIB) (Scios dual beam, FEI, Portland, USA) technique. The relative densities of the as‐fabricated HEB samples were computed by comparing experimental densities obtained via Archimedes’ method using distilled water as the immersion medium with theoretical densities derived from the lattice constants obtained through Rietveld refinement of the crystal structure. The well‐polished surfaces of the as‐fabricated HEB were etched for 1 min by soaking in an acid mixture (HF: HNO_3_: H_2_O = 1:1:3) to enhance the visibility of grain boundaries. After etching, the surfaces were examined under an optical microscope to accurately assess the grain distribution and measure the grain sizes. At least 50 grains were counted to ensure the accuracy of the average grain size results. Moreover, the characterization of the as‐fabricated HEB samples by STEM was performed using a double Cs‐corrected TEM (Titan Themis G2, FEI, USA) equipped with a Super‐X EDS detector operating at 300 kV. HAADF‐STEM was employed to observe atomic structures directly, using a probe convergence angle of 25 mrad and a collection angle between 61 and 200 mrad. To accurately assess atomic displacements in the lattice structures of HEB samples, high‐resolution HAADF‐STEM images were corrected for drift distortion through an orthogonal image pair technique.^[^ ^65^ ^]^ Approximately 600 atomic site centers were identified in the STEM images using a 2D Gaussian fitting algorithm within the CalAtom software suite.^[^ ^66^ ^]^ Further refinement of atomic displacements was achieved via a displacement separation analysis, allowing for the direct extraction of atomic displacements from the lattice structure without prior assumptions, utilizing Fourier space filtering.^[^ ^67^ ^]^ Atomic‐resolution EDS mapping (400 × 400 pixels) of the samples was conducted in STEM mode, with a dwell time of 8 µs per pixel and a pixel size of 25.65 pm. The total acquisition time was ≈35 min, with the electron beam current maintained at ≈50 pA for both HAADF imaging and EDS mapping. Velox software (version 3.9) was used to generate raw EDS spectrum images and identify the characteristic X‐ray signals of elements, including Ti Kα, V Kα, Zr Kα, Nb Kα, Mo Kα, Hf Lα, and Ta Lα. The raw EDS maps were subsequently denoised using non‐local principal component analysis.

### Statistical Analysis

The results of the convergence tests in Figure S13 (Supporting Information) are presented as the mean ± standard deviation (SD).

## Conflict of Interest

The authors declare no conflict of interest.

## Author Contributions

Y.L. and Y.F. contributed equally to this work. Y.C. conceived and designed this work. Y.L. and H.Y. performed calculations. Y.F. and C.G. performed experiments. Y.C., H.Y., L. Z., and Y.L. analyzed the data and wrote the manuscript. All authors commented on the manuscript.

## Supporting information



Supporting Information

## Data Availability

Data sharing is not applicable to this article as no new data were created or analyzed in this study.
