# Supplementary material for: Lattice‐Distortion‐Driven Reduced Lattice Thermal Conductivity in High‐Entropy Ceramics
Source: Adv Sci (Weinh). 2025 Mar 20;12(19):2501157. doi: 10.1002/advs.202501157 (PMC12097022; doi:10.1002/advs.202501157)
Supplement: Supplementary file 1 — Supporting Information [file ADVS-12-2501157-s001.docx]

Supporting Information

Lattice-Distortion-Driven Reduced Lattice Thermal Conductivity in High-Entropy Ceramics

*Yiwen Liu, Yaming Fu, Fangchao Gu, Hulei Yu*, Lei Zhuang, Yanhui Chu**

Y. Liu, Y. Fu, F. Gu, L. Yu, L. Zhuang, Y. Chu

School of Materials Science and Engineering

South China University of Technology

Guangzhou, 510641, China

E-mail: huleiyu@scut.edu.cn (H. Yu); chuyh@scut.edu.cn (Y. Chu)


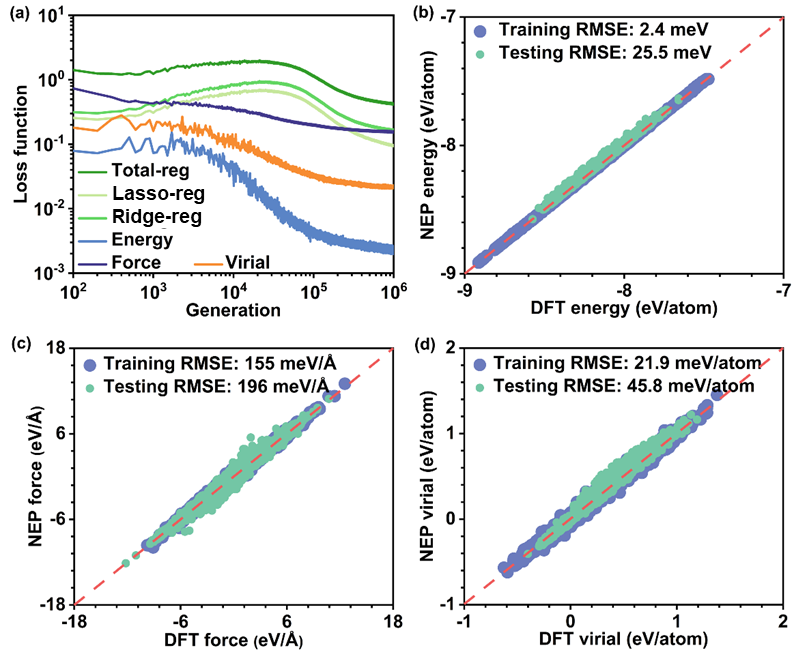


**Figure S1.** Performance of the constructed NEP based on unary and binary diboride training dataset. (a) Evolution of various loss functions with respect to generations. (b) Energy, (c) force, and (d) virial from NEP and DFT calculations for the unary and binary diboride training dataset and HEB testing dataset.


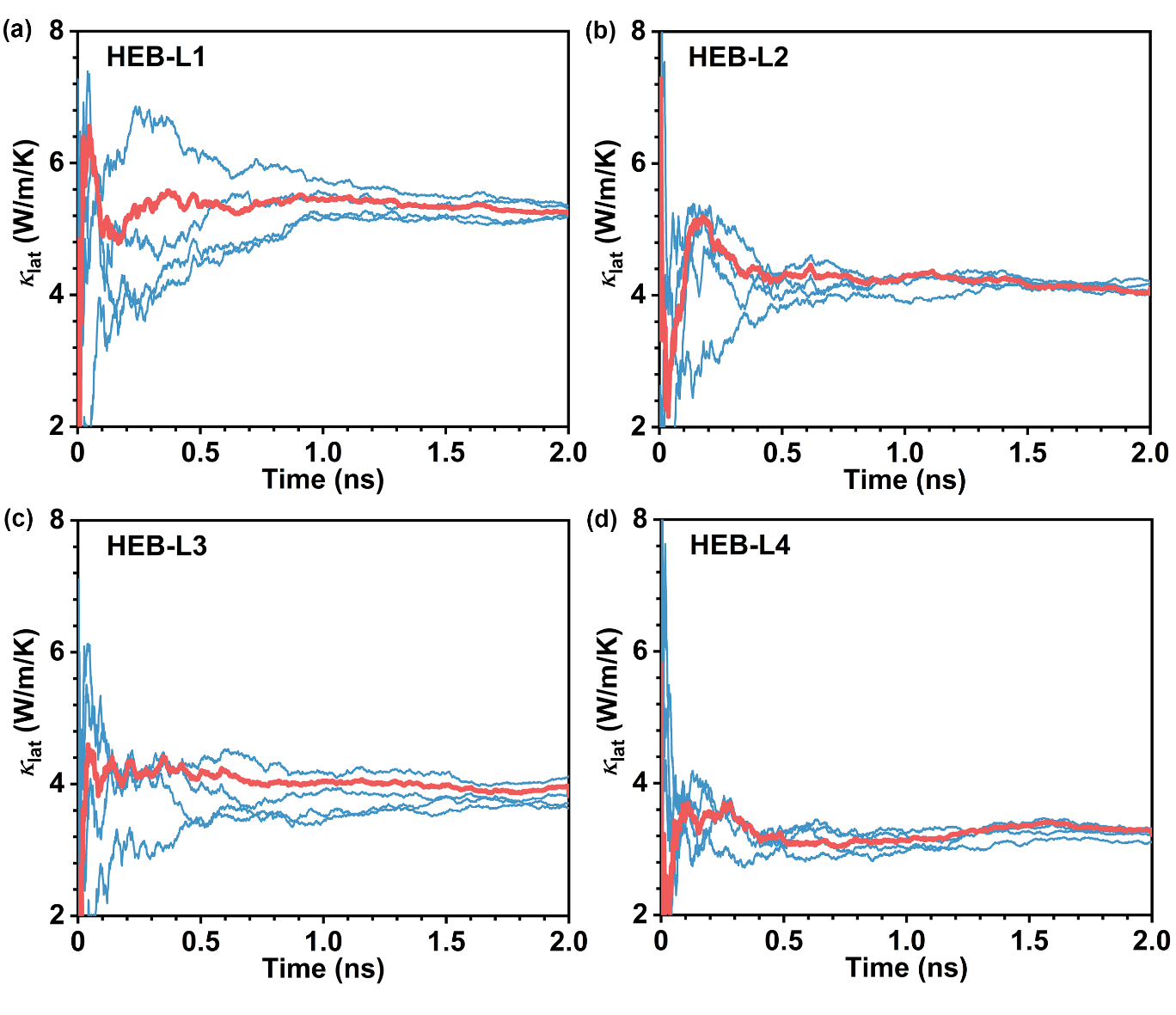


**Figure S2.**Calculated $\kappa_{\mathrm{lat}}$ as a function of correlation time for HEB-L1−L4.


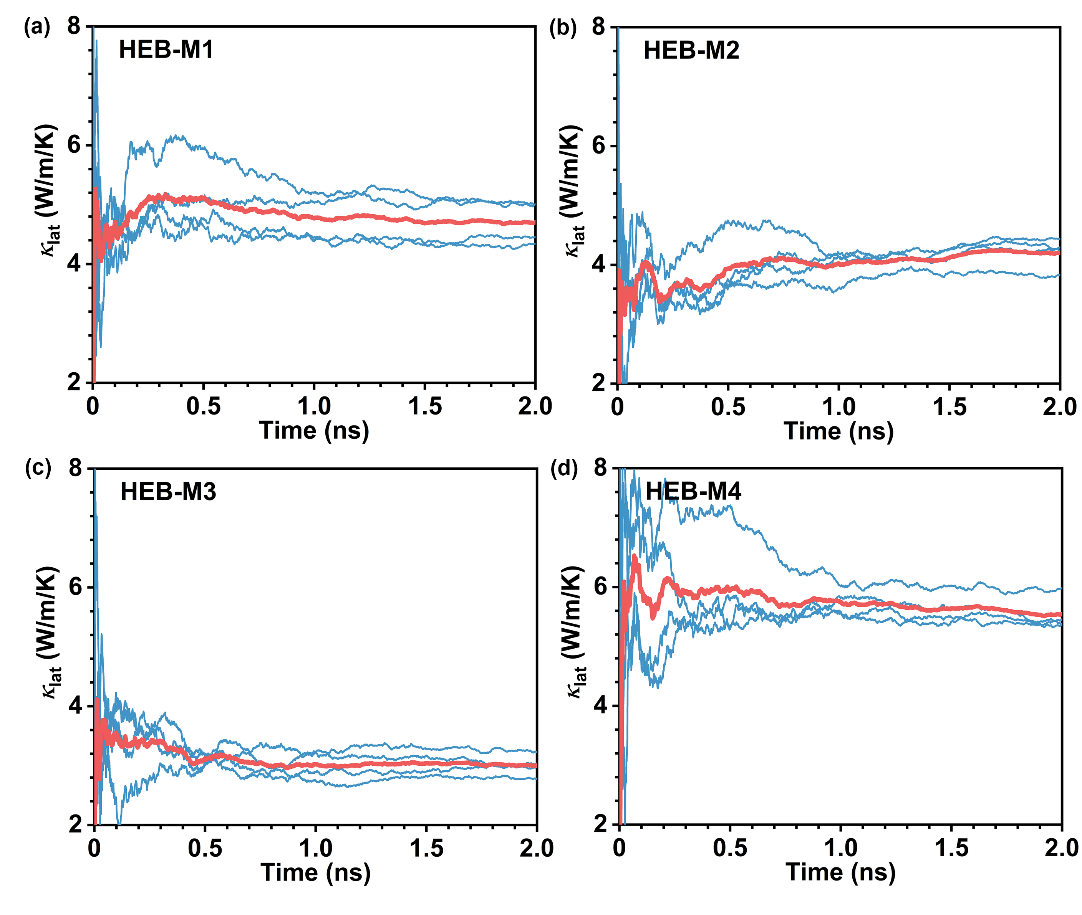


**Figure S3.** Calculated $\kappa_{\mathrm{lat}}$ as a function of correlation time for HEB-M1−M4.


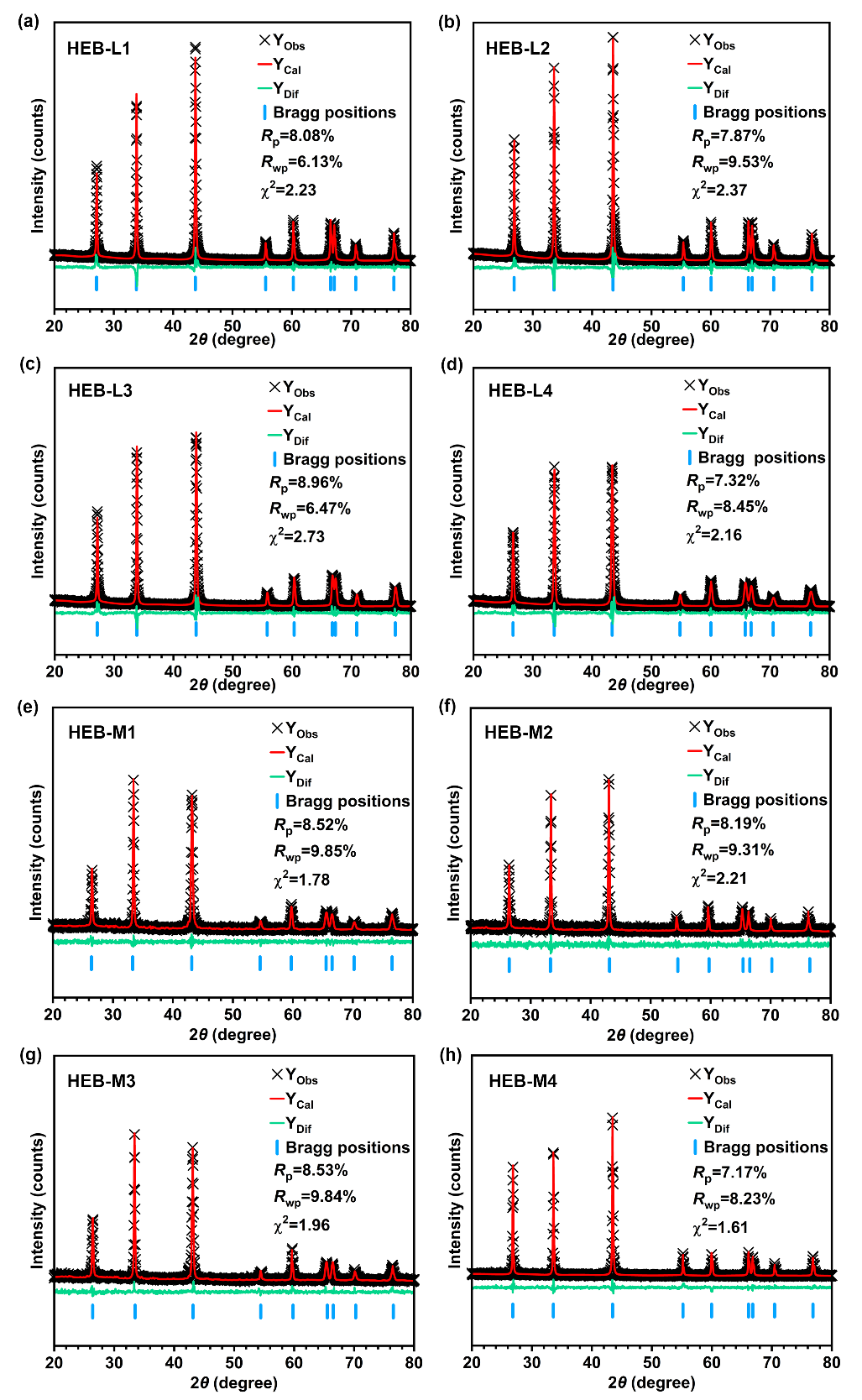


**Figure S4.** Rietveld refinement of XRD patterns of the as-fabricated HEB samples: (a) HEB-L1. (b) HEB-L2. (c) HEB-L3. (d) HEB-L4. (e) HEB-M1. (f) HEB-M2. (g) HEB-M3. (h) HEB-M4.


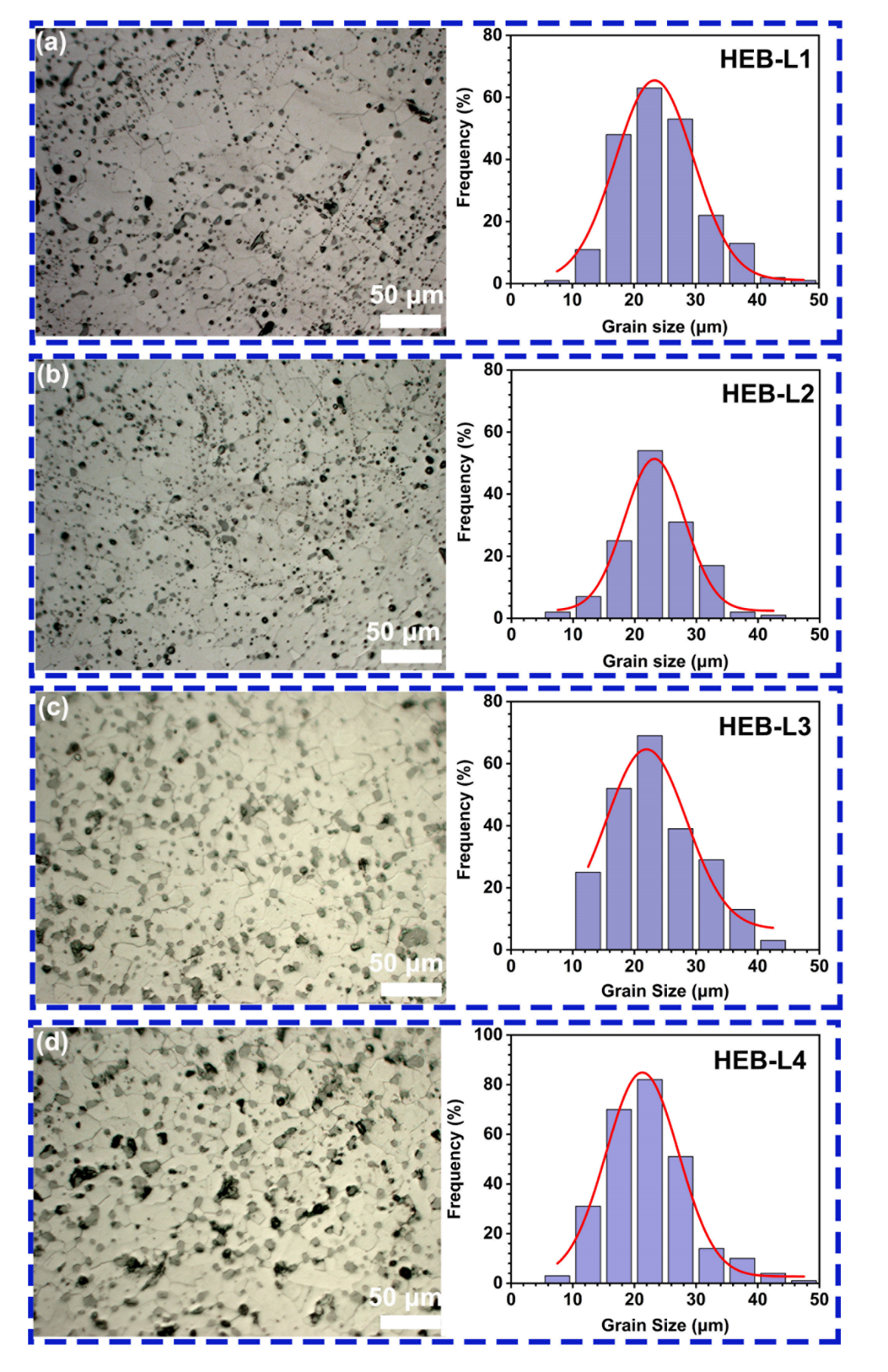


**Figure S5.** Optical microscope images and the corresponding grain size distribution of the as-fabricated HEB-L1−L4 samples.





**Figure S6.** Relative density and average grain size of the as-fabricated HEB-L1−L4 samples.


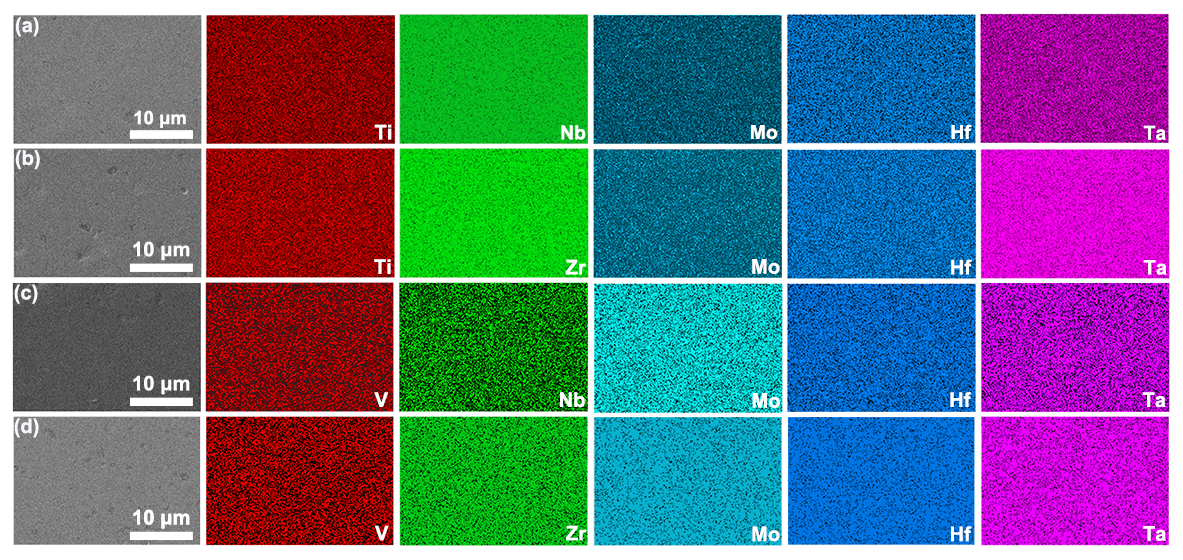


**Figure S7.** SEM image and corresponding EDS compositional maps of the as-fabricated HEB-L1−L4 samples. (a) As-fabricated HEB-L1 samples. (b) As-fabricated HEB-L2 samples. (c) As-fabricated HEB-L3 samples. (d) As-fabricated HEB-L4 samples.


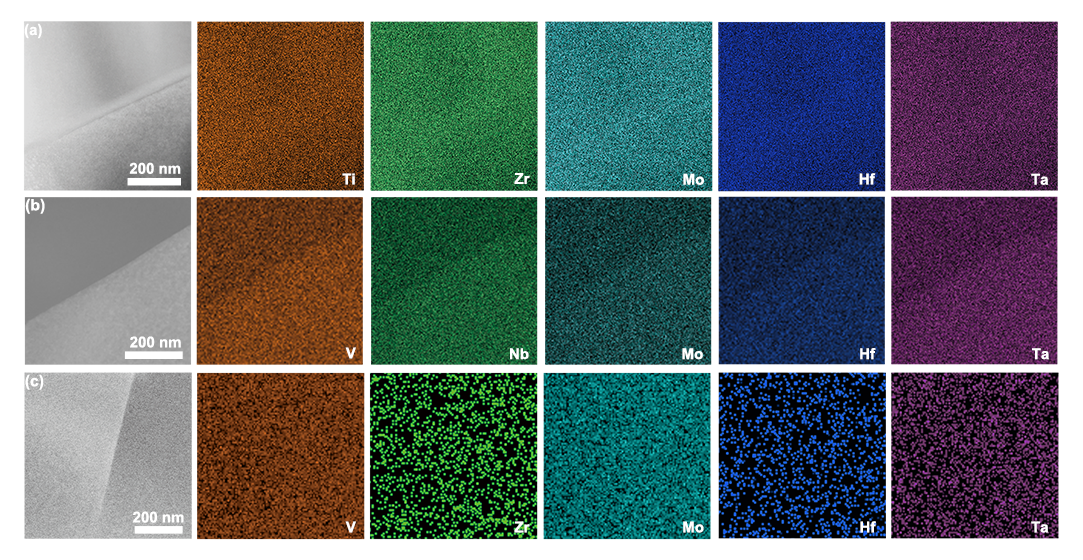


**Figure S8.** STEM image and corresponding EDS compositional maps of the as-fabricated HEB-L1−L4 samples. (a) As-fabricated HEB-L2 samples. (b) As-fabricated HEB-L3 samples. (c) As-fabricated HEB-L4 samples.

**
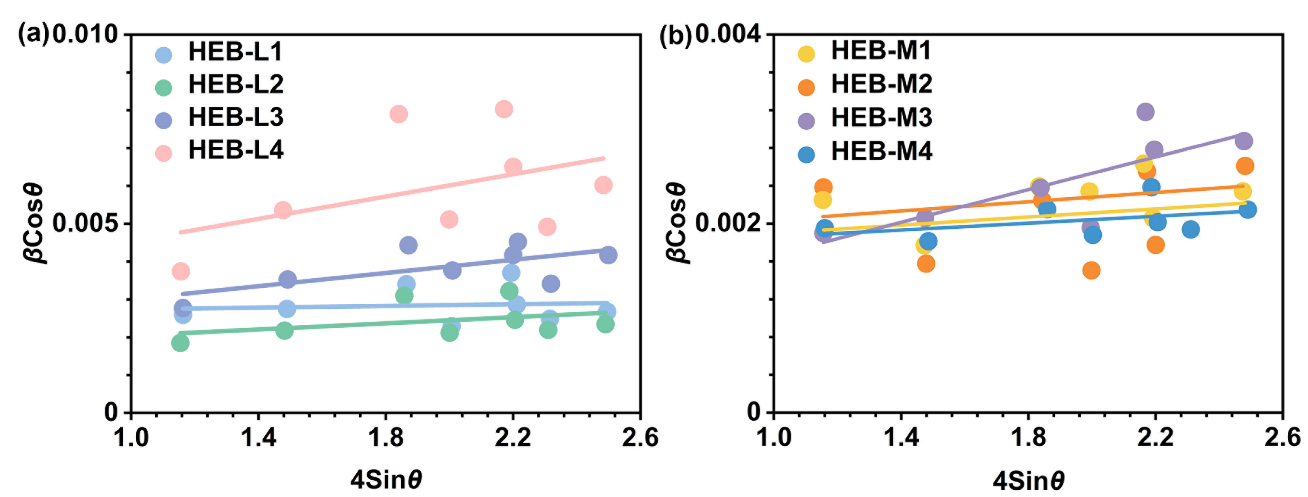
**

**Figure S9.** Detailed calculation process of *ε* based on Williamson-Hall analysis.





**Figure S10.** Measured thermal conductivities of the as-fabricated HEB-M1−M4 samples.


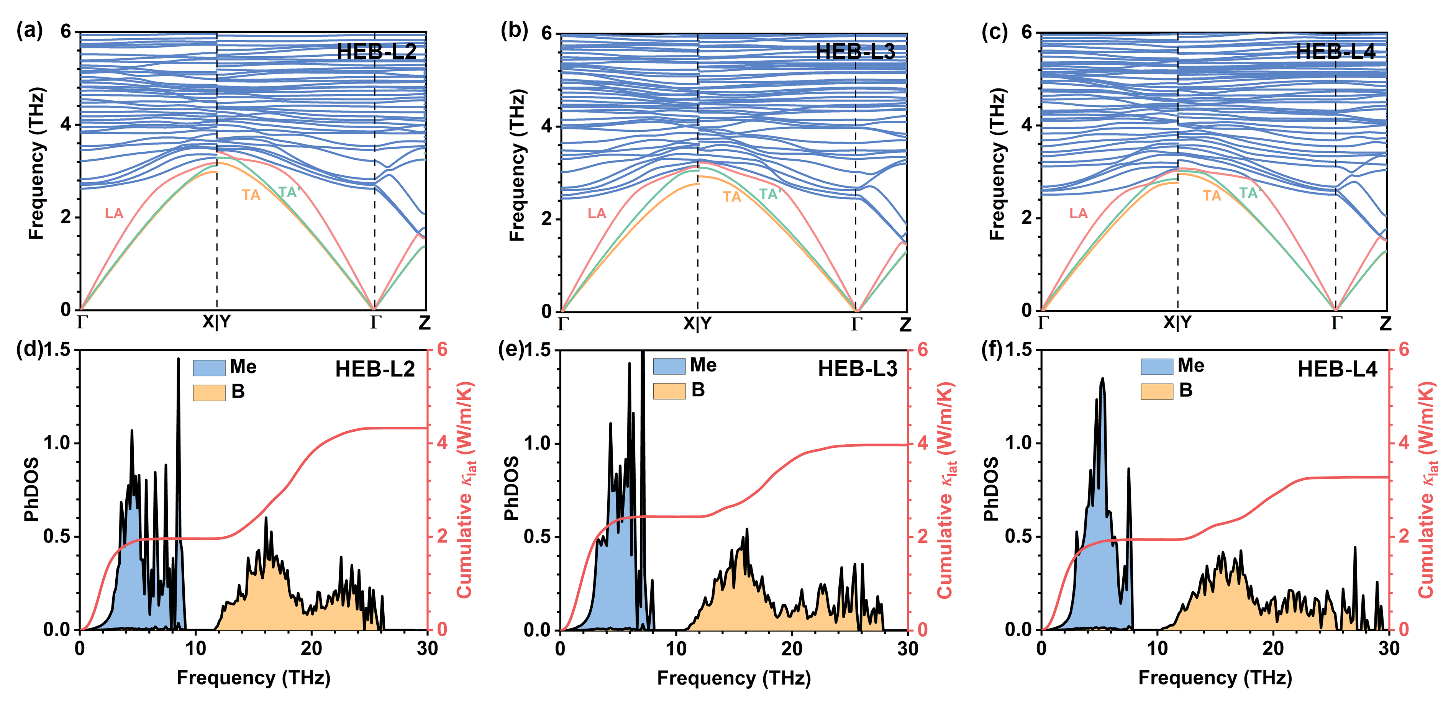


**Figure S11.** Phonon dispersion, PhDOS, and cumulative $\kappa_{\mathrm{lat}}$ for (a,d) HEB-L2, (b,e) HEB-L3, and (c,f) HEB-L4, respectively.





**Figure S12.** Volumetric and shear strain fluctuation of HEB-L1−L4.


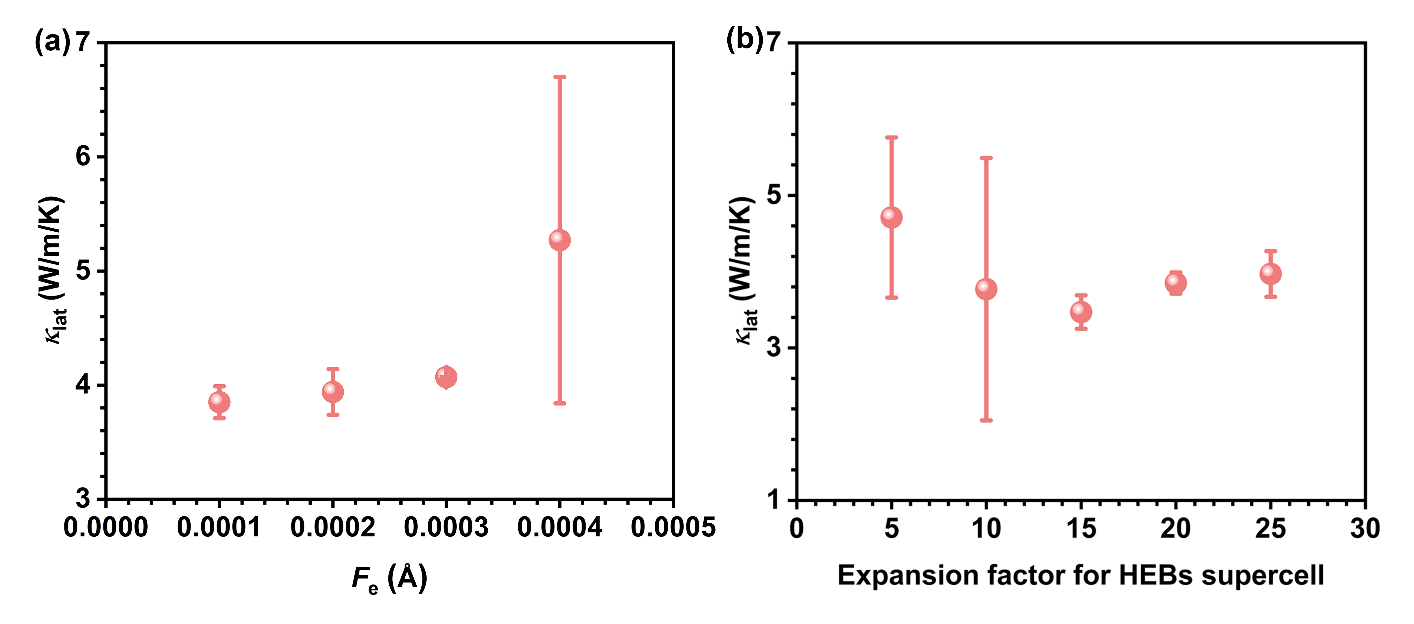


**Figure S13.** Convergence tests of (a) different driving forces and (b) size effects on the lattice thermal conductivity of HEB-L4.

**Table S1.** Compositions of 37 HEBs and the corresponding *U*, $\bar{M}$, and ${}_{m}$.

| Compositions | *U* (meV/atom) | $\bar{M}$ (Da) | ${}_{m}$ |
| --- | --- | --- | --- |
| (Ti_1/5_Nb_1/5_Mo_1/5_Hf_1/5_Ta_1/5_)B_2_ (HEB-L1) | 0.633 | 46.95 | 0.413 |
| (Ti_1/5_Zr_1/5_Mo_1/5_Hf_1/5_Ta_1/5_)B_2_ (HEB-L2) | 0.669 | 46.84 | 0.418 |
| (V_1/5_Nb_1/5_Mo_1/5_Hf_1/5_Ta_1/5_)B_2_ (HEB-L3) | 0.692 | 46.84 | 0.396 |
| (V_1/5_Zr_1/5_Mo_1/5_Hf_1/5_Ta_1/5_)B_2_ (HEB-L4) | 0.746 | 47.04 | 0.401 |
| (Ti_1/5_V_1/5_Nb_1/5_Mo_1/5_Hf_1/5_)B_2_ (HEB-M1) | 0.691 | 38.28 | 0.506 |
| (Ti_1/5_V_1/5_Zr_1/5_Nb_1/5_Ta_1/5_)B_2_ (HEB-M2) | 0.693 | 38.13 | 0.529 |
| (Ti_1/5_V_1/5_Zr_1/5_Nb_1/5_W_1/5_)B_2_ (HEB-M3) | 0.720 | 38.33 | 0.547 |
| (Ti_1/4_V_1/4_Nb_1/4_Ta_1/4_)B_2_ (HEB-M4) | 0.672 | 38.26 | 0.657 |
| (Ti_1/4_Nb_1/4_Mo_1/4_Ta_1/4_)B_2_ | 0.604 | 42.01 | 0.437 |
| (Ti_1/4_Zr_1/4_Nb_1/4_Ta_1/4_)B_2_ | 0.628 | 41.62 | 0.450 |
| (Ti_1/4_Nb_1/4_Mo_1/4_Hf_1/4_)B_2_ | 0.636 | 41.81 | 0.424 |
| (Ti_1/4_Zr_1/4_Mo_1/4_Ta_1/4_)B_2_ | 0.683 | 41.87 | 0.442 |
| (V_1/4_Nb_1/4_Mo_1/4_Ta_1/4_)B_2_ | 0.669 | 42.27 | 0.416 |
| (Ti_1/4_Zr_1/4_Nb_1/4_Hf_1/4_)B_2_ | 0.596 | 41.41 | 0.436 |
| (V_1/4_Zr_1/4_Nb_1/4_Ta_1/4_)B_2_ | 0.722 | 41.88 | 0.429 |
| (V_1/4_Nb_1/4_Mo_1/4_Hf_1/4_)B_2_ | 0.718 | 42.06 | 0.403 |
| (V_1/4_Zr_1/4_Mo_1/4_Ta_1/4_)B_2_ | 0.780 | 42.13 | 0.421 |
| (V_1/4_Zr_1/4_Nb_1/4_Hf_1/4_)B_2_ | 0.701 | 41.67 | 0.415 |
| (V_1/4_Zr_1/4_Mo_1/4_Hf_1/4_)B_2_ | 0.775 | 41.92 | 0.407 |
| (Ti_1/4_Nb_1/4_Hf_1/4_Ta_1/4_)B_2_ | 0.580 | 48.89 | 0.452 |
| (Ti_1/4_Mo_1/4_Hf_1/4_Ta_1/4_)B_2_ | 0.657 | 49.14 | 0.441 |
| (V_1/4_Nb_1/4_Hf_1/4_Ta_1/4_)B_2_ | 0.664 | 49.15 | 0.431 |
| (V_1/4_Mo_1/4_Hf_1/4_Ta_1/4_)B_2_ | 0.731 | 49.40 | 0.420 |
| (Ti_1/4_Zr_1/4_Hf_1/4_Ta_1/4_)B_2_ | 0.627 | 48.75 | 0.459 |
| (V_1/4_Zr_1/4_Hf_1/4_Ta_1/4_)B_2_ | 0.731 | 49.01 | 0.438 |
| (Ti_1/4_V_1/4_Nb_1/4_Hf_1/4_)B_2_ | 0.611 | 38.06 | 0.639 |
| (Ti_1/4_V_1/4_Zr_1/4_Ta_1/4_)B_2_ | 0.739 | 38.12 | 0.662 |
| (Ti_1/4_V_1/4_Zr_1/4_Hf_1/4_)B_2_ | 0.733 | 37.92 | 0.644 |
| (Ti_1/5_Zr_1/5_Nb_1/5_Hf_1/5_Ta_1/5_)B_2_ | 0.615 | 46.64 | 0.426 |
| (V_1/5_Zr_1/5_Nb_1/5_Hf_1/5_Ta_1/5_)B_2_ | 0.688 | 46.84 | 0.409 |
| (Ti_1/5_V_1/5_Zr_1/5_Nb_1/5_Hf_1/5_)B_2_ | 0.697 | 37.97 | 0.514 |
| (Ti_1/5_V_1/5_Nb_1/5_Mo_1/5_Ta_1/5_)B_2_ | 0.642 | 38.45 | 0.521 |
| (Ti_1/5_V_1/5_Zr_1/5_Mo_1/5_Hf_1/5_)B_2_ | 0.753 | 38.17 | 0.509 |
| (Ti_1/5_V_1/5_Zr_1/5_Mo_1/5_Ta_1/5_)B_2_ | 0.738 | 38.34 | 0.524 |
| (Ti_1/5_V_1/5_Zr_1/5_Hf_1/5_Ta_1/5_)B_2_ | 0.712 | 43.84 | 0.604 |
| (Ti_1/5_V_1/5_Mo_1/5_Hf_1/5_Ta_1/5_)B_2_ | 0.694 | 44.15 | 0.590 |
| (Ti_1/5_V_1/5_Nb_1/5_Hf_1/5_Ta_1/5_)B_2_ | 0.648 | 43.95 | 0.599 |

**Table S2.** Me atomic percentages of the as-fabricated HEB-L1−L4 samples characterized by SEM-EDS.

| Samples | Elements （at.%） | | | | | | |
| --- | --- | --- | --- | --- | --- | --- | --- |
|  | Ti | Nb | Mo | Hf | Ta | Zr | V |
| HEB-L1 | 19.45 | 20.98 | 20.10 | 19.59 | 19.88 | – | – |
| HEB-L2 | 18.71 | 19.67 | 17.94 | – | 22.36 | 21.32 | – |
| HEB-L3 | – | 20.74 | 18.63 | 21.58 | 20.84 | – | 18.21 |
| HEB-L4 | – | – | 19.45 | 20.99 | 21.73 | 20.54 | 19.03 |

**Table S3.** Me atomic percentages of the as-fabricated HEB-L1−L4 samples characterized by STEM-EDS.

| Samples | Elements （at.%） | | | | | | |
| --- | --- | --- | --- | --- | --- | --- | --- |
|  | Ti | Nb | Mo | Hf | Ta | Zr | V |
| HEB-L1 | 19.32 | 21.03 | 20.10 | 19.87 | 19.68 | – | – |
| HEB-L2 | 19.37 | 19.65 | 18.29 | – | 22.33 | 20.36 | – |
| HEB-L3 | – | 20.39 | 19.17 | 21.43 | 20.89 | – | 18.12 |
| HEB-L4 | – | – | 20.23 | 21.16 | 21.35 | 20.19 | 17.07 |

**Table S4.** Phonon velocities of HEB-L1−L4.

| *v* (×100 m/s) | | | | | |
| --- | --- | --- | --- | --- | --- |
| composition | LA | TA | TA' | Average | Elastic-tensor |
| HEB-L1 | 67.43 | 48.85 | 50.75 | 55.68 | 54.73 |
| HEB-L2 | 66.54 | 48.87 | 49.60 | 55.00 | 54.57 |
| HEB-L3 | 65.92 | 48.77 | 49.37 | 54.69 | 53.08 |
| HEB-L4 | 62.93 | 47.28 | 49.28 | 53.16 | 52.18 |
